# A Frequency-Dependent and Nonlinear, Time-Explicit Five-Layer Human Head Numerical Model for Realistic Estimation of Focused Acoustic Transmission Through the Human Skull for Noninvasive High-Intensity and High-Frequency Transcranial Ultrasound Stimulation: An Application to Neurological and Psychiatric Disorders

**DOI:** 10.3390/bioengineering12111161

**Published:** 2025-10-26

**Authors:** Shivam Sharma, Nuno A. T. C. Fernandes, Óscar Carvalho

**Affiliations:** Center for Micro-Electro Mechanical Systems (CMEMS), University of Minho, 4800-058 Guimarães, Portugal; shivam0671@gmail.com (S.S.); oscar.carvalho@dem.uminho.pt (Ó.C.)

**Keywords:** transcranial focused ultrasound simulation, COMSOL Multiphysics, non-invasive brain stimulation, wave propagation, nonlinearity, attenuation, acoustics, neurorehabilitation

## Abstract

Transcranial focused ultrasound is a promising noninvasive technique for neuromodulation in neurological and psychiatric disorders, but accurate prediction of acoustic transmission through the skull remains a major challenge. In this study, we present a five-layer numerical human head model that integrates frequency-dependent acoustic parameters with nonlinear time-explicit dynamics to realistically capture ultrasound propagation. The model explicitly represents skin, trabecular bone, cortical bone, and brain, each assigned experimentally derived acoustic properties across a clinically relevant frequency range (0.5–5 MHz). Numerical simulations were performed in the frequency domain and time-explicit to quantify sound transmission loss and focal depth under high-intensity and high-frequency stimulation. The results show the effect of frequency, radius of curvature, and skull thickness on maximum pressure ratio, focal depth, and focus zone inside the brain tissue. Findings indicate that skull geometry, particularly radius of curvature and thickness, strongly influences the focal zone, with thinner skull regions allowing deeper penetration and reduced transmission loss. Comparison of the frequency-domain model with the time-explicit model demonstrated broadly similar trends; however, the frequency-domain approach consistently underestimated transmission loss and was unable to capture nonlinear effects such as frequency harmonics. These findings highlight the importance of nonlinear, time-explicit modeling for accurate transcranial ultrasound planning and suggest that the proposed framework provides a robust tool for optimizing stimulation parameters and identifying ideal target zones, supporting the development of safer and more effective neuromodulation strategies.

## 1. Introduction

Neurological disorders present a formidable global health challenge, since they are the leading cause of disability and the second leading cause of death throughout the globe [1,2], imposing a substantial burden on human well-being. Globally, more than 3 billion people were living with a neurological condition in 2021, making neurological conditions the leading cause of ill health and disability worldwide [3]. In 2019, neurological disorders globally caused an estimated 10.06 million deaths and lost 349.22 million disability-adjusted life years (DALYs) [4]. The economic impact is profound; for instance, annual costs per patient in Europe range widely, up to EUR 30,000 for neuromuscular disorders annually [5], reflecting the direct healthcare and indirect productivity losses. While globally, the economic impact of neurological disorders, including lost productivity and healthcare costs, is estimated in the trillions of US dollars, it is disproportionately affecting resource-limited countries [6]. Depression and anxiety are costing the global economy approximately EUR 1 trillion yearly and represent a leading cause of disability worldwide [7]. Despite advances in pharmacological and psychotherapeutic interventions, a considerable subset of patients remains treatment-resistant [8], often requiring invasive procedures such as stereotactic ablative surgery or deep brain stimulation. These invasive treatments are associated with severe adverse effects, including epilepsy (up to 10%), transient cognitive impairment, personality changes (up to 10%), intracranial hematomas, and even death in rare cases, as well as irreversible changes to brain structure [9,10]. This necessitates the development of effective non-invasive interventions. Transcranial focused ultrasound (tFUS) is a non-invasive technique that serves as a therapeutic avenue for a broad range of neuromodulation, including tissue ablation and suppression or enhancement of neural activity, to alleviate symptoms or restore function [11]. tFUS transmits mechanical energy through the intact skull to target deep brain regions with remarkable precision, achieving millimeter-sized focal spots [12,13]. tFUS is further classified into high-intensity focused ultrasound (HIFU) and low-intensity focused ultrasound (LIFU) depending on the acoustic intensity at the focal area, biological effects, and medical applications [14]. Beyond neurological applications, the lack of effective non-invasive devices for other parts of the body (such as the knee) [15,16] has also motivated research into ultrasound-based therapies as potential alternatives to stimulate cartilage regeneration and improve joint health [17,18].

The efficacy of tFUS in modulating brain activities has been demonstrated across numerous animal models, including rodents [19,20,21], frogs [22], cats [23], rabbits [24], and monkeys [25]. LIFU has shown therapeutic potential in animal and human studies for conditions such as disorders of consciousness [26], epilepsy [27], Parkinson’s disease [28], Alzheimer’s disease [29], depression [30], dementia, and other mental or psychiatric disorders [31,32]. Its ability to modulate neurons without causing damage, typically at energy levels below 500 mW/cm^2^ spatial peak-temporal average intensity (Ispta) [14], further underscores its therapeutic promise. More critically, HIFU can achieve the tumor removal of the liver [33], kidney [34], prostate [35], brain [36], and breast [37], proving it to be a non-invasive tumor treatment technology. HIFU also helps in treating internal bleeding [38] through thermal ablation; the same phenomena are responsible for tumor treatment. Thus, tFUS is a promising tool for a diverse range of medical applications, from fundamental neuroscience research in tumor treatment to therapeutic interventions for neurological and psychiatric disorders.

The success rate of tFUS applications in the brain has been obstructed by the skull and the challenges associated with targeting and monitoring treatments [39]. Numerous studies have been conducted over the years to extensively investigate the skull’s impact on tFUS. This includes examining the effect of acoustic properties [40,41] and thickness and density [42,43,44] of the skull on tFUS. Furthermore, researchers have also contributed to the tFUS scientific literature through the implication of a skull phantom [45] and mapping the human skull geometry [46] to the numerical analysis. A few studies have also numerically simulated transcranial ultrasound nonlinear and transient propagation through a multilayer head model at high frequencies [47,48]. Despite prior research yielding specific findings, these investigations have not examined the impact of practical skull configuration (including cortical and trabecular regions) and near-accurate acoustic and physical properties of the skull regions, together, on a complex, realistic human head system [47,48,49].

This study aims to numerically investigate the effect of skull regions (cortical and trabecular), along with skin and brain muscle, included in a realistic multilayer human head model, on the challenges faced by tFUS. The novelty of the present work lies in the near-accurate mapping of the human head (into five layers) during practical tFUS treatment and the nonlinear and transient numerical tFUS simulation for frequencies ranging from 0.5 MHz to 5 MHz, considering the transient acoustic properties, sound diffusivity of each layer. This effect of skull regions is presented in two parts: the first part focuses on the effect of different skull thicknesses, depending on different lobes, and their distance from the transducer on tFUS at constant source frequency; the second part focuses on the effect of frequency, from LIFU to HIFU region, on the tFUS propagation. This part investigates the effect of frequency-dependent acoustic property, namely sound diffusivity, of the cranial layers on the accuracy of the focal area. This work also includes an organized description of the numerical method used in COMSOL, which is not available in the literature to the best of the authors’ knowledge.

### High-Fidelity Transcranial Focused Ultrasound Numerical Modeling

Finite element method (FEM) simulations have enhanced predictive precision, uncovering insights for complex geometries and anisotropic material properties, such as those found in the human skull, which would otherwise not be possible through experimentation [50,51,52]. Computational analysis using FEM can address the intracranial pressure fields, focal displacement, and dose under realistic skull conditions [50,51,52,53]. FEM-based platforms like COMSOL Multiphysics are particularly powerful due to their robust multiphysics coupling capabilities, allowing for the simulation of complex interactions between ultrasound and biological tissues, including critical thermal and mechanical effects.

Skull is the dominant source of amplitude loss and phase distortion for tFUS, and small thickness variations can shift peak pressures measurably, underscoring the sensitivity of intracranial dose to local bone morphology [51,53]. Accurate wave modeling thus requires heterogeneous, frequency-dependent acoustics and elastic coupling at skull interfaces; domain decomposition and coupled acoustic–elastic formulations have been advanced to treat soft tissue as compressible fluid and skull as a heterogeneous solid with appropriate absorption, aided by absorbing boundary layers to prevent spurious reflections [54]. COMSOL Multiphysics can bridge key research gaps by modeling skull-induced aberration, attenuation, and safety constraints while accelerating translation to individualized therapeutic delivery. Recent validation studies and reviews show that combining physics-based full-wave models with CT-derived skull properties improves focal prediction, parameter selection, and safety margins, enabling more precise and reliable neuromodulation protocols in humans and large animals [51,52,53,55].

Mathematical attenuation models derived from COMSOL-based simulations and phantom experiments capture exponential dependence on skull thickness with high fidelity (R^2^ ≈ 0.9996) and low errors versus measurements, offering compact predictors for dose correction and preliminary planning. Prior studies have used COMSOL to generate training data and parametric sweeps for attenuation models, then validated against hydrophone and phantom data, demonstrating strong agreement and utility for rapid dose estimation across skull thicknesses and incident angles. The FEM’s strength in complex geometries and material anisotropy complements FDTD/k-space solvers, particularly when co-simulating thermal effects, shear conversion, damping, and boundary conditions aligned with patient-specific anatomy [51,52,53,55].

A persistent gap is variability in amplitude transmission and local heating risks due to individual skull properties; state-of-the-art validation shows reliable focal location and shape prediction but highlights larger errors for absolute pressure, motivating improved attenuation mapping beyond simple CT-derived models. This article deals with the precise mapping of the absolute pressure and its respective variability in amplitude transmission, for a realistic multi-layer complex human head model with different skull thicknesses and source frequencies, through FEM analysis using COMSOL Multiphysics.

## 2. Transcranial Ultrasound Numerical Method

The transcranial ultrasound propagation numerical method, used in this work, was able to accurately determine the acoustic properties, such as frequency-dependent acoustic attenuation coefficient and sound diffusivity, of different biological tissues, which was experimentally and analytically validated in our previous work [55]. The numerical analysis is performed with the help of COMSOL Multiphysics 5.3, like the previous work.

### 2.1. Governing Equations for Acoustic Wave Propagation Model in the Frequency Domain

For many therapeutic ultrasound applications, particularly when nonlinear effects are negligible or the acoustic intensity remains within the small-signal regime, it is sufficient to model wave propagation using the frequency-domain formulation of the acoustic wave equation. This avoids the complexity of finite-amplitude models while still capturing the essential physics of scattering, reflection, and attenuation in biological tissues. The starting point is the Helmholtz equation [56,57,58,59,60], which describes the spatial distribution of the acoustic pressure field in the frequency domain through Equation (1):

(1) $$\nabla^{2}\left(r,\omega \right)+k^{2}p\left(r,\omega \right)=0$$

where $p\left(r,\omega \right)$ is the complex acoustic pressure at position r and angular frequency ω. The wavenumber is defined through Equation (2):

(2) $$k=\frac{\omega }{c}+i\alpha$$

with c  (m/s) representing the speed of sound in the medium and α (Np/m) denoting the frequency-dependent attenuation coefficient. The inclusion of the imaginary component in k allows the Helmholtz formulation to account for thermoviscous and relaxation-related absorption effects that are prominent in soft biological tissues.

Unlike the nonlinear Westervelt framework, which incorporates harmonic generation and shock formation, the Helmholtz equation assumes linear propagation, time-harmonic excitation, and spatially homogeneous medium properties. These assumptions are valid when wave amplitudes are sufficiently small such that higher-order nonlinearities are negligible. The frequency-domain approach is particularly advantageous for steady-state or harmonic excitation problems, as it reduces the governing partial differential equation to an elliptic form suitable for numerical methods such as the finite element method (FEM) or boundary element method (BEM).

Thus, the present work employs the frequency-domain Helmholtz equation to define acoustic fields under the assumptions of linear propagation, weak attenuation, and time-invariant medium parameters.

### 2.2. Governing Equations for Nonlinear Acoustic Wave Propagation Model

For high-intensity focused ultrasound (HIFU) applications, traditional small-signal assumptions often fail, as finite-amplitude wave effects become significant [56]. These effects lead to nonlinear distortion that accumulates with distance, ultimately resulting in harmonic generation and potential shock formation [61]. The Westervelt equation represents a fundamental approach for modeling such nonlinear phenomena when cumulative effects dominate local nonlinearities [62]. The nonlinearity is consistent in biological tissues, which exhibit reflection, rarefaction, scattering, and attenuation of waves. Therefore, the present work employs the Westervelt equation [63] to define acoustic fields for nonlinear wave propagation incorporating thermos-viscous losses presented in Equations (3) and (4).

(3) $$\nabla^{2}p-\frac{1}{c^{2}}\frac{\partial^{2}p}{\partial t^{2}}+\frac{\delta }{c^{4}}\frac{\partial^{3}p}{\partial t^{3}}+\frac{\beta }{\rho c^{4}}\frac{\partial^{2}p^{2}}{\partial t^{2}}=0$$

(4) β=1+B/(2A)

This equation is the extension of the classical wave equation for finite amplitude waves with propagation distance greater than the wavelength [64]. The first two terms represent the variation of p (Pa), total acoustic pressure, with respect to *t* (s), time, due to mechanical perturbations. Attenuation or dissipation is frequency-dependent and is considered in the third term, with δ (m^2^/s) representing sound diffusivity accounting for viscous and thermal dissipation. The right-hand side of Equation (1) describes the nonlinear propagation of the acoustic wave, with β (dimensionless) being the nonlinearity parameter, which quantifies the effect of nonlinearity on the local speed of sound in the fluid, due to which phenomena like higher harmonics and wave distortion are experienced. In the above equations, ρ (kg/m^3^) is the density of the medium, c (m/s) is the speed of sound (SoS) in the medium, and B/A (dimensionless) is the nonlinearity coefficient [55,65].

The Westervelt equation implementation assumes several key conditions that ensure the model’s validity for progressive wave propagation, where cumulative nonlinear effects surpass local nonlinearities [66]. These include the following conditions: 1. weak nonlinearity (second-order effects dominate higher-order terms); 2. time-invariant medium properties (material parameters remain constant during propagation); 3. quiescent initial conditions (the material is initially at rest); 4. cumulative dominance (nonlinear effects accumulate over distances much greater than one wavelength). The well-known Westervelt equation, considering all the assumptions, is expressed by Equation (5). The nonlinearity of the medium restricts the use of constant SoS throughout the spatial domain. Therefore, c is replaced with $c_{0}$ [56], local or quiescent SoS in the medium quantified by the parameter of nonlinearity expressed in Equation (6).

(5) $$\frac{1}{\rho c_{0}^{2}}\frac{\partial^{2}p}{\partial t^{2}}-\nabla \cdot \left(\frac{1}{\rho }\left(\nabla p-q_{d}\right)+\frac{\delta }{\rho c_{0}^{2}}\frac{\partial \nabla p}{\partial t}\right)=\frac{\beta }{\rho^{2}c_{0}^{4}}\frac{\partial^{2}}{\partial t^{2}}\left(p^{2}\right)+Q_{m}$$

(6) $$c=c_{0}+\left(\beta -1\right)u$$

here u (m/s) is the acoustic particle velocity defined locally, and $q_{d}$ and $Q_{m}$ are dipole and monopole sources, respectively, that might be inserted in the medium.

### 2.3. Numerical Implementation on COMSOL Multiphysics

The implementation of the frequency-dependent domain in COMSOL Multiphysics utilizes the inhomogeneous Helmholtz equation, transforming Equations (1) and (2) into Equations (7) and (8).

(7) $$\nabla \left(-\frac{1}{\rho_{c}}\left(\nabla p_{t}-q_{d}\right)\right)-\frac{{k_{eq}}^{2}p_{t}}{\rho_{c}}=Q_{m}$$

(8) $${k_{eq}}^{2}=\frac{\omega^{2}}{{c_{c}}^{2}}-{k_{z}}^{2}$$

Compared to the classical Helmholtz equation, which assumes a homogeneous medium with constant density and no explicit sources, Equations (7) and (8) introduce several important generalizations. First, the Laplacian operator is reformulated to include the spatial variation in density $\rho_{c}$, allowing the model to account for heterogeneity in the propagation domain where material properties are not uniform. Second, source terms are explicitly incorporated: the monopole term $Q_{m}$ represents isotropic point sources of acoustic pressure, while the dipole term $q_{d}$ captures directional forcing and secondary source effects within the medium. Third, the simple wavenumber definition in Equation (2) is replaced by the equivalent wavenumber in Equation (8), allowing distinction of the transverse and axial components of wave propagation, which is particularly relevant in layered or anisotropic media such as biological tissues. Together, these modifications extend the Helmholtz framework from an idealized, source-free model to one capable of describing realistic acoustic fields in complex, inhomogeneous domains.

In this formulation, attenuation is formulated by utilizing an equivalent fluid model, in which the term $\rho_{c}$ and $c_{c}$ is given by Equations (9) and (10),

(9) $$\rho_{c}=\rho {\left(1+\frac{i\omega \delta }{c^{2}}\right)}^{-1}$$

(10) $$c_{c}=c{\left(1+\frac{i\omega \delta }{c^{2}}\right)}^{-1}$$

that incorporates an equivalent-fluid model that mimics the propagation of sound in a fluid, including losses due to thermal conduction and viscosity in the bulk of the fluid through the use of a sound diffusivity coefficient (δ, m^2^/s)

The simulation of nonlinear ultrasound propagation in COMSOL Multiphysics is based on the nonlinear pressure acoustics equations [67] with a time-dependent propagation factor [68], derived from the second-order Westervelt equation. The well-known Westervelt equation, expressed by Equation (5), is solved in conservative form [69] of nonlinear pressure acoustic equations in a time-explicit numerical method in this work, expressed in Equations (11) and (12).

(11) $$\frac{1}{\rho c_{0}^{2}}\frac{\partial p}{\partial t}+\nabla \cdot \left[1+\frac{\beta p}{\rho c_{0}^{2}}\right]u=Q_{m}$$

(12) $$\rho \frac{\partial u}{\partial t}+\nabla \cdot \left(pI\right)=q_{d}$$

This equation is implemented through the ‘General Dissipation’ fluid model under Nonlinear Pressure Acoustics, Time Explicit (NATE) package of COMSOL Multiphysics (Version 5.3). This allows for capturing the attenuated pressure waves by defining losses through the sound diffusivity. Spatial and temporal discretization is achieved by discontinuous Galerkin FEM (dG-FEM), using high-order polynomial basis functions (typically quartic, or 4th order), and an explicit time stepping scheme, respectively. The optimal mesh size for the quartic element, as per dG-FEM implementation, follows the relationship in Equation (13) [70]:

(13) $$\Delta x\approx \frac{c_{0}}{6N_{0}f_{0}}$$

where $N_{0}$ represents the number of harmonics to resolve and $f_{0}$ is the fundamental frequency. Furthermore, the explicit time-stepping scheme maintains stability through the Courant–Friedrichs–Lewy (CFL) condition represented in Equation (14) [71]:

(14) $$\mathrm{CFL}=\frac{c_{0}\Delta t}{\Delta x}<0.2$$

for quadratic elements, ensuring numerical stability throughout the simulation.

## 3. Materials and Methods

### 3.1. Material Properties and Human Head Topography

**Figure 1:**
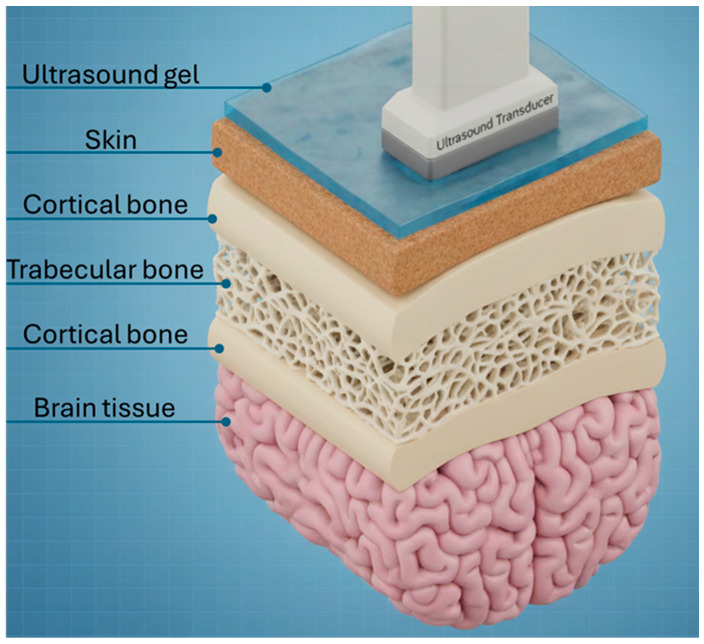
Three-dimensional (3D) representative cross-section schematic of tFUS through the skull to reach the brain in a human head. It is possible to see the representative layer since the transducer, a thin layer of ultrasound gel in contact with the skin, the trabecular bone with a density, followed by the representation of the highly porous cortical bone, and the following cortical bone, until it finally reaches the represented part of the brain.

A 5-layer model to estimate the maximum pressure transmission through a human head is proposed. A total of 5 impedances is used to represent gel, skin, skull, blood, and brain tissues (Figure 1). The properties are presented in Table 1, validated by Fernandes et al. [55], which are used as the input parameters for the numerical analysis of the human skull. Sos (c), density (ρ), and the nonlinearity coefficient (B/A) for all the elements of the human head model are invariant with respect to time and source frequency. The brain tissues, such as gray matter and white matter, have properties with negligible differences; thus, their distribution throughout the brain is assumed to be a single brain tissue, for simplification in considering different regions of the human head, in this work, and an average value for all the properties is considered.

The nonlinearity of the human head, which makes it a complex model to resolve for acoustic pressure distribution, in the past, was the attenuation caused by different parts of the human head. The attenuation, which includes reflection, scattering, and absorption, due to the nonlinear biological tissues found inside the human head and skull-induced aberration, is considered in the COMSOL Multiphysics numerical method through the sound diffusivity (δ) values of the respective elements. The attenuation is dependent on frequency, and so is the sound diffusivity, presented in Table 2.

Aquasonic gel properties [72] are considered in this work, as it is widely used in diagnostic and therapeutic medical ultrasound procedures. The B/A value of the ultrasound gel is preferred to be closer to the respective value of the skin and lower than hydrogels [73]. All the gel properties used in this work are within the acceptance criteria [74] of the commercially used ultrasound gel set by the International Commission on Radiation Units and Measurements Report-61, Tissue Substitutes, Phantoms, and Computational Modeling in Medical Ultrasound. Therefore, the absorption coefficient for the gel is selected as 7 dB/cm/MHz. The fitting parameter for the frequency dependency of the attenuation coefficient of polymeric gel is nearly equal to 1, and the experimental result by Remo et al. [75] showed a slight change in attenuation coefficient with respect to frequency. Therefore, the absorption coefficient is consistent with frequency, and the sound diffusivity is calculated by Equation (15), by Hamilton et al. [63], only for the gel:

(15) $$\delta =\frac{2c_{0}^{3}\alpha }{\omega^{2}}$$

here α(1/m) is the absorption coefficient and ω(1/s) is the angular frequency. Whereas the sound diffusivity for the biological tissues is calculated by Fernandes et al. [55] through the experimentally validated frequency-dependent Equation (16):

(16) $$\delta (f)=\delta_{0}f^{n}$$

where δ(f) is the sound diffusivity at frequency *f* (MHz), $\delta_{0}$ is the reference diffusivity constant at *f* = 1 MHz, and *n* is the frequency exponent, a material-dependent parameter calculated through numerical simulation and validated experimentally. The sound diffusivity (δ) for all the model domains used in this work is presented.

Furthermore, this work also considers different regions of the human head, namely the temporal, frontal, occipital, and parietal. Different regions are considered by including different thicknesses of the human skull, trabecular and cortical bone, into the model. Table 3 presents the values of the different thicknesses of the skull reported by Na and Wang [76].

The 5-layer model in this work breaks down the skull thickness into two layers of dense cortical bone (outer and inner tables) sandwiching a porous trabecular (diploë) bone layer. The outer cortical layer is thicker than the inner cortical layer, with a median thickness ratio of about 1.68 between the outer and inner cortical layers [77]. The value of cortical thickness to trabecular thickness is considered to be 3.4:1 for all the regions considered in this work, for the simplification of our model, and to maintain consistency. This value is selected by averaging the mean approximated value for the total cortical-to-trabecular thickness ratio obtained by Boruah et al. [77], summarized in Table 4.

The overall skull contour exhibits continuous changes in curvature, and a uniform radius for the entire skull would cause inaccuracies in acoustic and mechanical modeling, as documented by both imaging and morphometric studies [78,79]. Studies on brachycephalic skulls show local curvatures described by circular arcs [80], meaning that distinct small parts of the skull can be approximated to a local curvature. Therefore, the radius of curvature of the skull can be approximated locally for small sections [81], of a particular region, commensurate with the transducer of surface diameter ~7.4 cm, similar to the ones used in our experimental validation study [55]. Thus, for accurate modeling, different radii of curvature (RoC) were considered for different skull regions, for the 5-layer human head simulations, presented in Table 5.

### 3.2. Simulation Setup (COMSOL Multiphysics)

#### 3.2.1. Geometry

**Figure 2:**
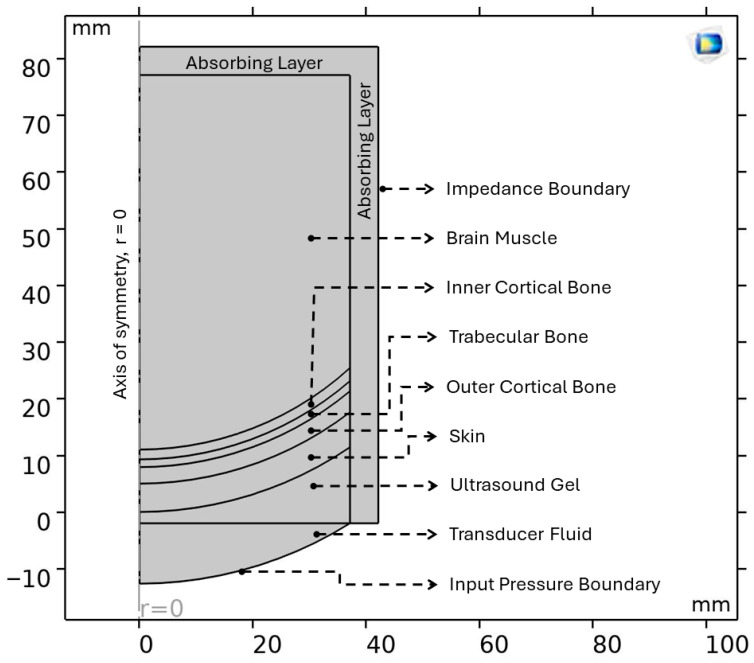
Axisymmetric human head model setup in COMSOL Multiphysics 5.3, where it is possible to observe each domain and its boundaries.

A 5-layer human head model for each part of the human head is simplified by considering a section with curvature, as shown in Figure 1. This geometry is implemented in this work as 2D-axisymmetric to optimize computational cost by minimizing the total degree of freedom provided to the solver. The modifications to Figure 1 that are made to the 2D-axisymmetric geometry, used in the numerical model, are as follows: (i) all biological tissue layers and gel layers are uniform and compact, i.e., no inclusion of air molecules inside the layer. Specifically, the trabecular layer and brain muscles included in the numerical model are not used in the same way as presented in Figure 1; (ii) all the layers are in close contact with each other without any air gaps between interfaces; (iii) the effect of porosity in different layers is included in the material properties of the respective tissues. Figure 2 shows the configurational representation of the different layers implied in this work. The values for different skull thicknesses, the respective breakdown of skull thickness into outer cortical, trabecular, and inner cortical thickness, and the radius of curvature of the small section, are all presented in Table 6, which defines the dimensions of different geometries considered in this work. Figure 1 depicts the pictorial representation of the 5-layer realistic human head, described so far, that includes ultrasound gel, skin, trabecular bone sandwiched between two cortical bone layers, and the brain tissue.

#### 3.2.2. Boundary Conditions

The input signal waveform was applied as a pressure boundary condition that replicates the signal applied in the experimental procedure, validated and calibrated in our previous work [56]. The frequency domain considered in this work is double the maximum frequency of the experimental input signal, obtained through FFT. This approach ensures relevant harmonic components, generated by the nonlinearity in wave propagation, are simultaneously captured along with the fundamental wave frequency. Other boundary conditions include sound-hard (wall) conditions and impedance conditions on the exterior walls of the system under the condition, shown in Figure 2. These conditions are important to acoustically isolate the system under consideration.

An absorbing layer, in Figure 2, is considered inside the system domain, acting as a transition layer between the system under consideration and the exterior environment. This layer significantly absorbs or attenuates the acoustic wave, thus minimizing the artificial reflections. This is an indispensable part of any acoustic numerical simulation to obtain accurate results on wave propagation. Three probes were positioned, one near the source and the other two at the interfaces of skin–skull and skull–brain tissue. This allows for capturing the effect of skin and skull on the acoustic pressure attenuation during wave propagation. The transducer fluid is assumed to have properties similar to ultrasound gel for simplification of the simulation model. For simulations in the frequency domain, the absorbing layer was removed and replaced by perfectly matched layers.

**Figure 3:**
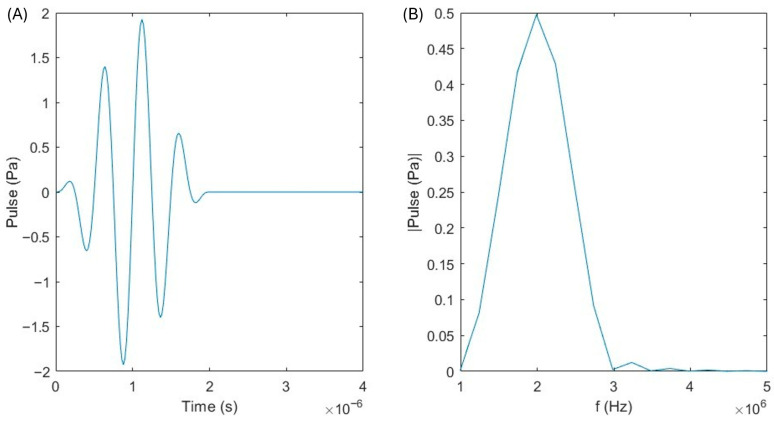
Input signal in the (**A**) time domain and (**B**) frequency domain for 2 MHz, where it is possible to observe the central frequency of 2 MHz in the frequency spectrum.

For the time-explicit simulation, a single ultrasonic pulse was employed to model acoustic wave propagation, as is displayed in Figure 3. This pulse was defined with a finite duration and controlled amplitude, corresponding to the experimentally chosen low-frequency excitation and constant transducer pressure used by Fernades et al. [55]. By using a single pulse rather than a continuous wave, the simulation captures the transient behavior of the acoustic field, including wavefront propagation, reflection, and attenuation within the medium.

In the simulation, the initial conditions were set such that the acoustic pressure p=0 and its time derivative $\frac{\partial p}{\partial t}=0$ throughout the computational domain. These conditions correspond to quiescent fluid at rest, with no pre-existing motion or acoustic perturbations. By starting from this state, the simulation ensures that all subsequent pressure waves are solely generated by the applied ultrasonic pulse, allowing for an accurate assessment of wave propagation, attenuation, and focusing dynamics within the medium.

#### 3.2.3. Mesh and Solvers

Triangular mesh elements were employed throughout the domain, whereas the mesh size depends on Equations (5) and (6). The CFL conditions play an important role in the design of mesh element size for nonlinear wave propagation. The CFL values considered in this work are 0.2 for soft tissues and gel, and 0.1 for the skull domain, namely, trabecular and cortical bone. Using a uniform CFL condition, hence the mesh density, could not solve the nonlinear partial differential equation in the relatively higher acoustic attenuation region of the domain. The mesh elements generated throughout the system, during different frequency simulations, are presented in Table 7.

The Runge–Kutta method (RK34) was employed for time stepping with a constant time interval to maintain numerical stability. The simulation duration was rigorously calibrated to coincide with the time needed for the input signal to completely traverse the sample, thus preventing superfluous computations beyond the wave’s transit interval.

The Multifrontal Massively Parallel Sparse direct solver (MUMPS) solver is employed with a memory allocation factor of 1.2 to provide sufficient memory resources for managing huge sparse matrices, while preventing overconsumption that can result in instability or excessive computational burden. This allocation achieves a balance between solver performance and memory efficiency, which is particularly crucial for simulations involving thin meshes and high-frequency wave propagation in heterogeneous media.

#### 3.2.4. Post Processing

Post-processing of the simulation results was conducted using MATLAB R 2024b (MathWorks, Natick, MA, USA), which provided a flexible and robust environment for data analysis and visualization. The primary quantities of interest were the sound transmission loss (STL) and the focal pressure distribution, which were extracted from the time-explicit and frequency-domain simulations.

The STL was calculated based on the acoustic pressure measured at the transducer surface and at the focal point, using the following relationship in Equation (17):

(17) $$\mathrm{STL}\;=\;20\log\left(\frac{p_{transducer}}{p_{focus}}\right)$$

where $p_{transducer}$ is the pressure at the transducer probe and $p_{focus}$ is the pressure at the focal zone. This formulation provides a direct measure of the acoustic energy transmission through the medium, accounting for attenuation, scattering, and reflection effects.

MATLAB scripts were employed to extract the pressure field data from the simulation outputs, compute the STL, and visualize both the spatial distribution of the acoustic field and the temporal evolution of the pressure at the focus.

The post-processing workflow ensured reproducibility and facilitated comparison between different simulation conditions, providing a clear quantitative framework for analyzing the effectiveness of transcranial focused ultrasound in both time-explicit and frequency-domain contexts.

## 4. Results and Discussion

### 4.1. Pressure Acoustics—Frequency Domain Study

**Figure 4:**
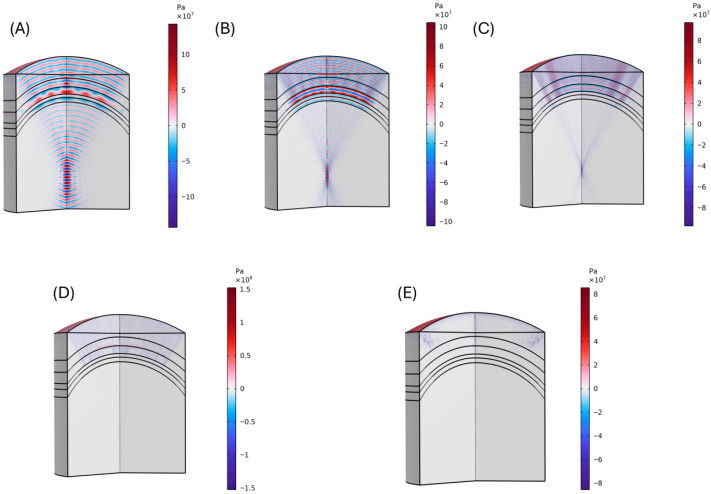
Frequency domain acoustic wave propagation through the human head for (**A**) 0.5 MHz, (**B**) 1 MHz, (**C**) 2 MHz, (**D**) 4 MHz, and (**E**) 5 MHz. It is possible to observe that as frequency increases, the focus becomes narrower, but due to the biological tissues’ inherent high attenuation, the depth the signal reaches reduces drastically.

Simulations conducted in the frequency domain enable the identification of focal regions and provide insight into whether the system has reached a steady-state response, as illustrated in Figure 4.

From the simulations in Figure 4, it is clear that as the excitation frequency increases, the acoustic pressure amplitude drops significantly while the focal zone becomes narrower and more localized. At 0.5 MHz (Figure 4A), the pressure field is strong, with a broad focal region and deeper penetration into the brain tissue. At 1–2 MHz (Figure 4B,C), the focal spot sharpens, but attenuation through the skull reduces the transmitted pressure considerably. By 4–5 MHz (Figure 4D,E), the transmitted pressure is dramatically diminished, and the focal region is much smaller, highlighting the strong frequency-dependent attenuation of bone. Another noticeable detail is the increased reflection and scattering patterns at higher frequencies, especially near skull boundaries, which further reduce the efficiency of acoustic energy transmission into the brain. This demonstrates the fundamental trade-off in transcranial ultrasound: lower frequencies penetrate better but yield less precise focusing, while higher frequencies provide tighter focal zones but suffer from strong attenuation and reduced intracranial pressure.

In order to figure out how the human anatomy would affect acoustic transmission, skull radius, skull thickness, and pressure change were studied.

**Figure 5:**
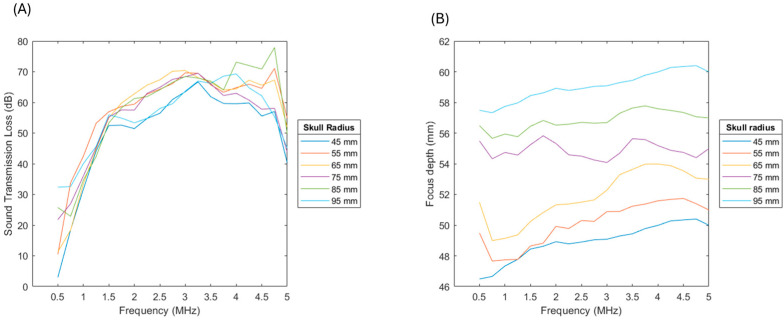
Sound transmission loss and focus depth results with respect to the RoC of the skull and frequency variation. It is possible to observe that (**A**) sound transmission loss and (**B**) focus depth both increase with an increase in skull radius.

In Figure 5A, we can see that sound transmission loss increases both with frequency and with larger skull radius, indicating that thicker or more curved skulls act as stronger barriers to ultrasound propagation. At lower frequencies (below 1 MHz), transmission loss remains relatively moderate across skull radii, suggesting that low-frequency ultrasound penetrates more effectively regardless of skull thickness. However, as frequency increases beyond 2–3 MHz, transmission loss becomes highly sensitive to skull radius, with steeper rises observed in thicker skulls. This aligns with the physics of wave propagation, where higher frequencies are more prone to scattering, absorption, and reflection within dense structures. Figure 5B complements this by showing that the focal depth of ultrasound is reduced with increasing frequency and skull radius, particularly evident at frequencies above 2 MHz. In these cases, the focus shifts closer to the skull, meaning that effective energy delivery into deeper brain regions becomes increasingly difficult. Together, the plots demonstrate that both skull geometry and operating frequency must be carefully balanced: smaller radii and lower frequencies favor transmission, while larger skulls and higher frequencies exacerbate losses and shift the focus away from the brain interior. This highlights why low-frequency ultrasound is generally favored for transcranial applications, despite its broader focal spot.

**Figure 6:**
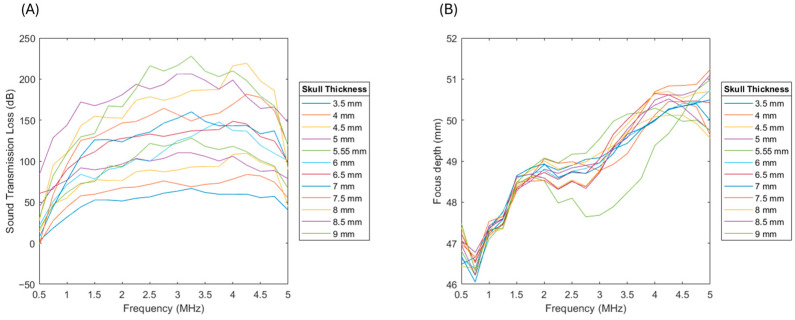
(**A**) Sound transmission loss and (**B**) focus depth results with respect to skull thickness and frequency variation, where an increase in sound transmission loss is observed with an increase in thickness.

In Figure 6A, the influence of skull thickness on sound transmission loss becomes evident when coupled with frequency. At low frequencies (<1 MHz), sound transmission loss remains relatively moderate across thickness values, suggesting that low-frequency ultrasound can penetrate even thicker skull regions with reasonable efficiency. However, as frequency increases, thicker skulls show a much greater attenuation, with losses exceeding 80–100 dB for skulls above 7–8 mm. This is consistent with the known physics of wave propagation: thicker bone leads to greater absorption, scattering, and phase aberrations, particularly at higher frequencies. Anatomically, this trend highlights why ultrasound transmission is more feasible through naturally thinner skull regions such as the temporal bone (~2–4 mm), compared to the occipital or parietal bones, which can exceed 6–8 mm in thickness. In Figure 6B, the focus depth is also influenced: as both frequency and skull thickness increase, the focus shifts shallower, moving closer to the skull surface rather than penetrating deeper into the brain. For thin temporal regions, the focus can remain at depths of ~50 mm, making them favorable windows for transcranial applications. In contrast, thicker frontal or occipital regions reduce penetration depth, complicating targeted neuromodulation or therapy. These findings reinforce the anatomical dependency of ultrasound efficacy: thinner skull regions facilitate deeper, more efficient energy delivery, while thicker areas introduce strong transmission losses and degrade focal accuracy, making low-frequency sonication essential for accessing deeper brain structures in those regions.

**Figure 7:**
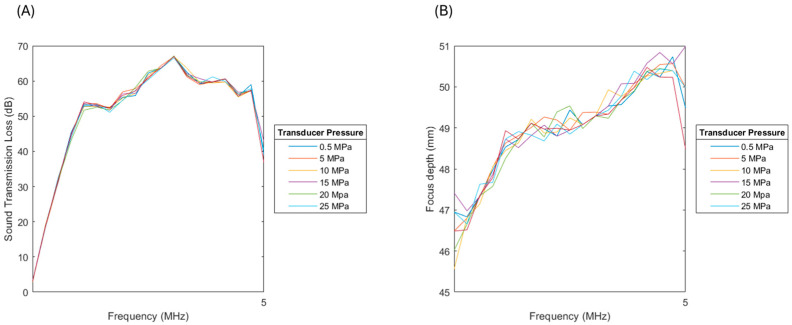
(**A**) Sound transmission loss and (**B**) focus depth results with respect to maximum source amplitude and frequency variation, where it is possible to observe that transducer pressure has a minimum effect on sound transmission loss and focus depth.

The transducer pressure appeared to exert minimal influence on both STL and the focal depth. As illustrated in Figure 7A, only minor fluctuations are observed in STL with varying transducer pressures; overall, an increase in applied pressure does not lead to a measurable decrease in STL. This observation is consistent with the underlying physics, since STL is defined as the ratio of the acoustic signal at the transducer to that measured at the focal zone, making it relatively insensitive to moderate variations in transducer contact pressure. Similarly, Figure 7B shows that the focal depth exhibits only slight increases with increasing transducer pressure. This effect can be explained by the marginal improvement in acoustic coupling and wave propagation through the medium, which allows the ultrasound energy to reach slightly deeper regions. However, these changes are not significant within the examined pressure range. Overall, while the transducer pressure is critical for defining the absolute acoustic pressure at the focus, ensuring reproducibility and safety in experimental setups, it does not substantially alter STL or the focal depth, indicating that the system is robust to small variations in applied transducer force.

Based on the parametric analysis, the optimal experimental conditions were selected for subsequent simulations. Specifically, a low-frequency excitation in combination with a constant transducer pressure was chosen for modeling acoustic wave propagation in the time-explicit framework. This choice reflects the observed trends in STL and focal depth, where low frequencies provided favorable transmission characteristics, and variations in transducer pressure had a negligible impact. By fixing the pressure, the model ensures a reproducible and controlled absolute pressure at the focus, while low-frequency excitation maximizes wave penetration and minimizes attenuation through the cranial medium.

### 4.2. Pressure Acoustics—Time-Explicit Study

**Figure 8:**
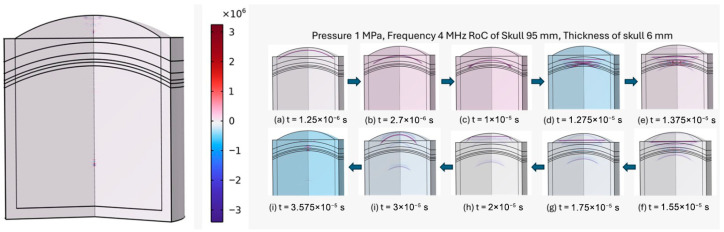
Time-explicit simulation of ultrasonic wave propagation showing the formation of the focal zone.

The time-explicit simulations provide a detailed view of the transient behavior of ultrasonic wave propagation through the medium. By applying a single pulse under a constant transducer pressure, these simulations capture the evolution of the acoustic pressure field over time, allowing for precise assessment of focal zone formation, wavefront propagation, and attenuation, as can be seen in Figure 8. Unlike frequency-domain analyses, which represent steady-state responses, the time-explicit approach enables the visualization of dynamic phenomena, including reflections, interference, and the temporal buildup of pressure at the focus. At the time of maximum pressure ratio observed in the brain tissue, the region for which the signal pressure is more than 5% of the maximum pressure is considered the focus zone in this work.

Figure 8 illustrates the propagation of the ultrasonic wave through the medium as captured by the time-explicit simulation. The wavefront emanates from the transducer and travels through the biological tissues, gradually converging toward the focal region. A short frequency analysis of the obtained signal can be seen in Supplemental Material Figure S2. As the wave propagates, constructive interference leads to an increase in pressure magnitude, clearly delineating the focal zone. The figure highlights the spatial localization of the maximum pressure, confirming that the pulse effectively concentrates acoustic energy at the intended focus. The Minor reflections and diffractions along the propagation path can also be observed, but they do not significantly affect the formation of the focal zone.

**Figure 9:**
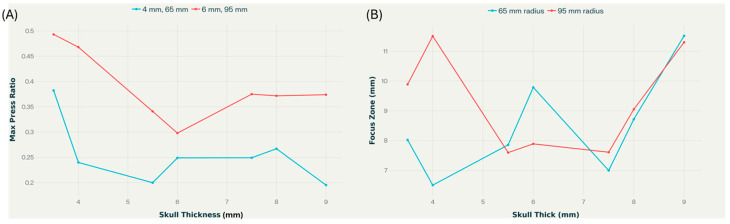
Dependence of maximum pressure ratio (**A**) and focus zone (**B**) on skull thickness, for 65 mm and 95 mm skull radius of curvatures, at 2 MHz source frequency and 1 MPa pressure amplitude.

Figure 9 depicts how transcranial ultrasound transmission properties change with increasing skull thickness at constant frequency (2 MHz) and source pressure (1 MPa) for two radii of curvature (65 mm and 95 mm). The maximum pressure ratio exhibits a modest decreasing trend as thickness increases for both radii, with higher values consistently observed for the 95 mm curvature. In contrast, the focus zone demonstrates nonmonotonic behavior, with increasing skull thickness, particularly for thinner skulls. For a given frequency (2 MHz) and pressure (1 MPa), the 95 mm curvature generally yields a wider focus zone than the 65 mm, except at intermediate thicknesses where both curves converge. The observed non-monotonic behaviors arise from complex wave interactions at skull-tissue interfaces. Mode conversion at skull boundaries transforms longitudinal waves into shear waves, which subsequently reconvert to longitudinal waves upon transmission, creating multiple wave groups with different arrival times and amplitudes [82,83]. Constructive and destructive interference between direct transmitted waves and skull-reverberated waves produces oscillatory pressure patterns, influencing focus quality [84]. The skull’s heterogeneous microstructure and varying porosity generate frequency-dependent scattering effects that interact nonlinearly with thickness changes [43,47]. These mechanisms do not scale linearly with anatomical parameters, explaining why optimal transmission occurs at intermediate rather than extreme values. Understanding these interactions is crucial for patient-specific treatment planning in transcranial ultrasound applications.

The findings highlight that thinner skulls and larger curvatures favor higher pressure transmission and more controlled focal regions. Notably, extreme values in the focus zone occur at both thin and thick skulls, highlighting the complex interplay between anatomical and acoustic parameters when targeting precise focal regions in transcranial ultrasound applications.

**Figure 10:**
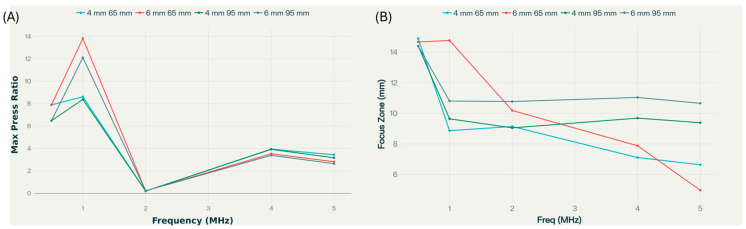
Dependence of maximum pressure ratio (**A**) and focus zone (**B**) on source frequency, with pressure amplitude 1 MPa, for skull thicknesses 4 mm and 6 mm, and the RoC of the skull 65 mm and 95 mm.

The maximum pressure ratio (Figure 10) shows that the maximum pressure ratio is highest at lower frequencies (0.5–1 MHz) for all skull thicknesses and radii of curvature but drops dramatically at 2 MHz before partially recovering at 4–5 MHz, especially for thinner skulls. The effect is more pronounced with thicker skulls, but for both thicknesses, the 65 mm radius configuration yields slightly higher transmission values at lower frequencies than the 95 mm, though this reverses near 4 MHz. The 6 mm skull with 65 mm curvature demonstrates a pronounced peak at 1 MHz, suggesting a strong dependency of transcranial ultrasound transmission on both bone thickness and the radius of curvature. For all cases, thicker skulls (6 mm) generally yield higher pressure ratios at 1 MHz, but the advantage diminishes at higher frequencies, where transmission efficiency becomes more similar across geometries.

The focus zone figure shows that zone width tends to decrease as frequency increases, especially from 0.5 MHz to 5 MHz, regardless of skull thickness or curvature. Interestingly, lower frequencies yield larger and less tightly focused zones, which might be less desirable for focal targeting but can increase the effective treatment volume. Conversely, higher frequencies produce smaller focus zones but suffer from poorer pressure transmission.

The results emphasize the importance of patient-specific parameter optimization (frequency, skull thickness, transducer geometry) for maximizing both energy delivery and focal control in therapeutic or research-oriented transcranial ultrasound applications. The findings highlight that: 1. Lower frequencies enhance both pressure transmission and result in broader focal zones, making them suitable for situations where deep penetration and energy delivery are prioritized over tight localization. 2. Skull thickness significantly diminishes both the maximum pressure ratio and the focus zone at higher frequencies, underlining the challenge of individualized parameter selection for patient-specific applications. The anomaly in the results trend is insignificant in front of the non-monotonic behavior observed due to skull thickness variation. Thus, the complex interplay of reflection, refraction, and mode conversion is not highly nonlinear, as observed in skull thickness variation, and also, as further presented, in radius of curvature variation.

**Figure 11:**
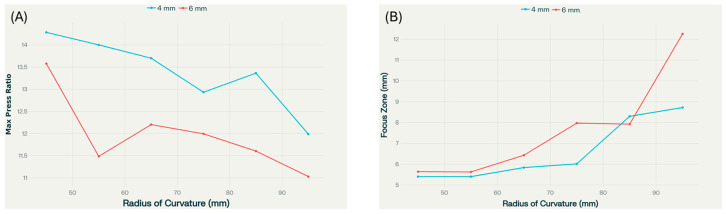
Dependence of (**A**) maximum pressure ratio (a) and (**B**) focus zone on radius of curvature of the skull for 4 mm and 6 mm skull thickness at 2 MHz source frequency, and 1 MPa pressure amplitude.

Figure 11 shows a slight decrease in pressure transmission as the radius of curvature increases for both 4 mm and 6 mm skull thicknesses. The 4 mm skull consistently yields a higher maximum pressure ratio compared to the 6 mm skull, emphasizing the beneficial effect of thinner bone on acoustic energy delivery. The rate of decrease is gradual after 65 mm RoC value, with the lowest values observed at the largest radii. The focus zone chart reveals that focus zone width tends to increase as radius increases, particularly beyond 75 mm, and this effect is most pronounced for the 6 mm skull. For smaller radii (<75 mm), the focus zone remains nearly constant, but it widens sharply with further increases in radius of curvature. Thinner skulls enable higher pressure transmission across all radii of curvature. Thus, larger radii of curvature lead to broader focus zones but reduced maximum pressure ratios, especially in thicker skulls. For applications requiring high energy transmission and a tightly focused beam, smaller radii and thinner skulls offer clear advantages. The observed behaviors result from intricate acoustic interactions at the interfaces between cranium and tissue. As skull curvature varies, multiple physical mechanisms contribute simultaneously: acoustic impedance mismatch between skull bone (Z ≈ 4.7 MRayls) and surrounding tissues (Z ≈ 1.5 MRayls), which creates substantial reflection coefficients that fluctuate with geometric changes [43]. Additionally, refraction effects due to varying skull curvature alter wave propagation paths, particularly affecting focus zone geometry.

The clinical translation of these findings is particularly relevant for the individualized planning of transcranial ultrasound neuromodulation and ablation therapies. Since the results demonstrate strong dependence of transmission and focal parameters on skull thickness, curvature, and acoustic diffusivity, clinical implementation should incorporate patient-specific skull parameter extraction. This can be achieved through preoperative CT or MRI-based acoustic mapping, where skull density ratio, cortical/trabecular segmentation, and local curvature are quantified to derive the acoustic impedance and attenuation coefficients used in numerical models. Recent studies show that CT Hounsfield units can be linearly correlated with ultrasound speed and density, allowing for conversion models that automatically generate acoustic property maps for use in FEM or k-space simulations [85,86,87,88]. Such individualized modeling enables pre-treatment planning, optimizing sonication frequency, focal depth, and transducer curvature for each patient while minimizing off-target exposure. Integration of this framework into clinical workflows, potentially through automated segmentation and parameter extraction pipelines, could substantially enhance treatment precision, safety, and reproducibility in both tFUS neuromodulation and HIFU ablation settings.

There are potential limitations to the model setup and the numerical model used in this work, anticipated by the authors. The 2D-axisymmetric geometry limits the actual asymmetric skull geometry representation and the transducer misalignments during clinical trials. As with any numerical method, there exists a potential for systematic errors to arise. The possible origins of these inaccuracies include the application of a direct solver in the study and the discretization technique employed in the finite element analysis. A direct solver simultaneously resolves all equations, which may lead to the accumulation of errors due to computational approximations of the solution. Moreover, second-order discretization, specifically quadratic Lagrangian elements, may influence the mesh quality, as the discretization depends on the approximation accuracy between adjacent components.

Another key limitation of the present work is that the proposed five-layer human head model has not yet been experimentally validated through in vitro or in vivo measurements, although an initial validation can be found in Supplemental Material Figure S1. Future studies should aim to perform quantitative experimental verification of the simulated pressure transmission and focal characteristics using human skull phantoms or ex vivo skull specimens under comparable sonication conditions. Such validation is essential to confirm the predictive accuracy of the model across different skull geometries, densities, and frequencies. Nevertheless, the results presented here show strong concordance with previously reported experimental and hybrid numerical–experimental studies. For example, Pichardo et al. (2011) demonstrated an exponential dependence of transmission loss on skull thickness and density across frequencies of 0.2–1 MHz using excised human skulls [43], while Clement & Hynynen (2002) reported similar focal distortion and pressure attenuation trends in experimental measurements through human cranial bone [89]. More recent validation efforts by Marquet et al. (2009) confirmed that finite-element and full-wave simulation frameworks using CT-derived skull maps accurately predict focal shift, transmission loss, and harmonic generation within 10–15% of measured values [90]. These agreements reinforce the reliability of the present model as a realistic computational platform for future patient-specific treatment planning and parameter optimization in tFUS and HIFU applications.

The findings highlight the following outcomes: 1. Lower frequencies enhance both pressure transmission and result in broader focal zones, making them suitable for situations where deep penetration and energy delivery are prioritized over tight localization. 2. Skull thickness significantly diminishes both the maximum pressure ratio and the focus zone at higher frequencies, underlining the challenge of individualized parameter selection for patient-specific applications. 3. Optimizing the balance between frequency, skull anatomy, and transducer curvature is essential for maximizing the efficacy and safety of transcranial ultrasound therapy or stimulation in neurological and psychiatric disorder treatments.

## 5. Conclusions

This study investigated the propagation of focused ultrasound through a medium using both frequency-domain and time-explicit simulations, with particular emphasis on the influence of transducer pressure and excitation frequency. The results demonstrate that transducer pressure has minimal effect on both sound transmission loss (STL) and focal depth, confirming that variations in applied force primarily affect absolute pressure at the focus rather than the efficiency of energy transmission. Low-frequency excitation was identified as optimal for maximizing wave penetration and focal energy concentration, while a constant transducer pressure ensured reproducibility and accurate assessment of absolute focal pressures.

This study demonstrates the successful implementation and validation of a state-of-the-art complex brain phantom and corresponding numerical simulation framework for investigating transcranial focused ultrasound. The combined computational approaches highlight the effects of varying ultrasound parameters, human anatomy, and material properties on acoustic wave propagation. In particular, the results demonstrate that an increase in skull radius and skull thickness increases STL and decreases focus depth, while transducer pressure has minimal effect on both STL and focal depth, confirming that variations in applied force primarily affect absolute pressure at the focus rather than the efficiency of energy transmission. Low-frequency excitation was identified as optimal for maximizing wave penetration and focal energy concentration, while a constant transducer pressure ensured reproducibility and accurate assessment of absolute focal pressures. Additionally, variations in wave frequency and the thickness of materials within the anatomy significantly influence transmission and focal localization, providing insight into the interplay between ultrasound parameters and heterogeneous tissue structures.

Slight discrepancies observed between the two numerical predictions are attributed to the simplified representation of tissue properties in the frequency domain and inherent uncertainties in their values. Despite these differences, the nonlinear, transient simulations offer critical insights that go beyond classical frequency-domain modeling, emphasizing the necessity of full spatiotemporal analysis for high-frequency transcranial ultrasound applications. These findings underscore the importance of considering nonlinear wave effects when designing and optimizing neuromodulation protocols, particularly in regions of the skull with higher acoustic transparency.

The study also outlines practical implications for ultrasound protocol development, including the design of waveforms that maximize transmission efficiency while maintaining safety, and the identification of skull regions suitable for energy delivery. Looking forward, future research can expand the model to full 3D patient-specific geometries based on MRI segmentation, incorporate thermal simulations, and explore cavitation thresholds to enhance the predictive capability and clinical relevance of the system. Current limitations include the use of static tissue properties and the absence of individualized anatomical modeling.

Overall, the results validate the utility of the complex numerical system, demonstrate the value of nonlinear transient simulations for understanding acoustic propagation in heterogeneous media, and provide a foundation for optimizing focused ultrasound protocols for safe and effective neuromodulation.

## Figures and Tables

**Table 1:** Density (ρ), Sos (c), and the nonlinearity coefficient (B/A) of different domain elements considered in the 5-layer human head model.

HIFU Model Domains	Density (ρ) (kg/m^3^)	SoS (*c*) (m/s)	Nonlinearity Coefficient (*B*/*A*) (Dimensionless)
Gel	1020	1580	7
Skin	1131	1655	7.2
Cortical Bone	1862	3194	13
Trabecular Bone	572	2325	80.6
Brain Tissues	1035	1550	7.3

**Table 2:** Sound diffusivity (δ) (m^2^/s) for different frequencies, considered in this work, of different domain elements considered in the 5-layer human head model.

HIFU Model Domains	Sound Diffusivity (δ) (m^2^/s)				
	0.5 MHz	1 MHz	2 MHz	4 MHz	5 MHz
Gel	0.005595	0.001399	0.00035	8.74 × 10^−5^	5.59 × 10^−5^
Skin	0.0046541	0.0026709	0.001542	0.000897	0.000446
Cortical Bone	0.1680300	0.0835210	0.0438240	0.0223810	0.0112840
Trabecular Bone	0.0165340	0.0123610	0.0094581	0.0075485	0.0071822
Brain Tissues	0.00337525	0.0017176	0.0008808	0.0004524	0.0003635

**Table 3:** Skull thickness for different regions of a normal human head by Na and Wang [76].

Human Head Regions	Male Skull Thickness (mm)	Female Skull Thickness (mm)
Frontal Central	8.1	7.4
Frontal Back	6.3	5.7
Parietal	5.9	5.6
Temporal	3.9	3.4
Occipital	9.3	8.3

**Table 4:** Variations in cortical and trabecular thicknesses for multiple anatomical regions of the calvarium, summarized from the results of Boruah et al. [77].

Region	Outer Cortical Thickness (O.Ct.Th, mm)	Trabecular Thickness (Tb.Th, mm)	Inner Cortical Thickness (mm) (I.Ct.Th, mm)	Approximate Total Cortical-to-Trabecular Ratio
Frontal	~1.2–1.5	~0.5–1.0	~0.6–0.9	2.4–4.8
Parietal	~1.0–1.3	~0.7–1.2	~0.5–0.8	1.8–3
Temporal	~1.0–1.2	~0.4–0.9	~0.5–0.7	2.1–4.8
Occipital	~1.3–1.6	~0.5–1.0	~0.6–0.9	2.5–5

**Table 5:** Values of the radius of curvature (RoC) obtained from different literature.

Skull Region	RoC (mm)	Notes	Reference
Frontal	44–97	Most curved region	Pirouzmand et al. [79]
Parietal	54–97	Flatter than frontal	Pirouzmand et al. [79]
Temporal	65–97	Approximated by mapping studies	Tolhuisen et al. [81]
Occipital	47–75	Comparable to parietal	Pirouzmand et al. [79]

**Table 6:** Dimensions used for the development of different geometries representing different regions of the human head model under source frequencies of 0.5, 1, 2, 4, and 5 MHz, and a source maximum pressure amplitude of 1 MPa.

Human Skull Region	Skull Thickness (mm)	Outer Cortical Thickness (Ct.Th, mm)	Trabecular Thickness (Tb.Th, mm)	Inner Cortical Thickness (mm)	RoC (mm)
Frontal	6	2.82	1.5	1.68	45, 55, 65, 75, 85, 95
Frontal	7.5	3.53	1.88	2.10	
Frontal	8	3.76	2	2.24	
Parietal	5.5	2.59	1.38	1.54	55, 65, 75, 85, 95
Parietal	6	2.82	1.5	1.68	
Temporal	3.5	1.65	0.88	0.98	65, 75, 85, 95
Temporal	4	1.88	1	1.12	
Occipital	8	3.76	2	2.24	45, 55, 65, 75
Occipital	9	4.23	2.25	2.52	

**Table 7:** Total mesh elements used in each domain for different frequency simulations.

HIFU Model Domains	0.5 MHz	1 MHz	2 MHz	4 MHz	5 MHz
Gel	337	574	852	4214	7685
Skin	531	879	1932	7543	11,675
Cortical Bone	349	752	2098	4065	6170
Trabecular Bone	137	321	1197	1947	3033
Brain Tissues	2126	3165	12,101	47,921	75,031

## Data Availability

The original contributions presented in this study are included in the article/Supplementary Material. Further inquiries can be directed to the corresponding author.

## Supplementary Materials

The following supporting information can be downloaded at: https://www.mdpi.com/article/10.3390/bioengineering12111161/s1, Figure S1: Comparison of pressure distribution results from numerical model and experimentation using a layer of (a) skin, (b) cortical bone, (c) trabecular bone, and (d) brain muscle.; Figure S2: Signal response captured by the probe in the brain tissue at 45 mm depth in the time-domain at (A) 0.5 MHz, (B) 1 MHz, and (C) 2 MHz and in the frequency domain at (D) 0.5 MHz, (E) 1 MHz, and (F) 2 MHz, where it is possible to observe the higher frequency harmonics typical of biological tissue acoustic wave propagation.

## Abbreviations

The following abbreviations are used in this manuscript:

STL	Sound Transmission Loss
DOAJ	Directory of open access journals
TLA	Three-letter acronyms
LD	Linear dichroism
RoC	Radius of curvature
SoS	Speed of sound
