# Supplementary material for: A Frequency-Dependent and Nonlinear, Time-Explicit Five-Layer Human Head Numerical Model for Realistic Estimation of Focused Acoustic Transmission Through the Human Skull for Noninvasive High-Intensity and High-Frequency Transcranial Ultrasound Stimulation: An Application to Neurological and Psychiatric Disorders
Source: Bioengineering (Basel). 2025 Oct 26;12(11):1161. doi: 10.3390/bioengineering12111161 (PMC12649367; doi:10.3390/bioengineering12111161)
Supplement: Supplementary file 1 [file bioengineering-12-01161-s001.zip › bioengineering-3906777-supplementary.pdf]

Supplemental Material

Numerical Simulation Validation through Experimentation

The numerical method used in this work for transcranial ultrasound propagation has been validated in our previous work [59] in terms of pressure distribution and determining acoustic properties of the biological tissues, including skin, trabecular bone, cortical bone, and brain muscle. The result from the validation is presented below in Figure S1.

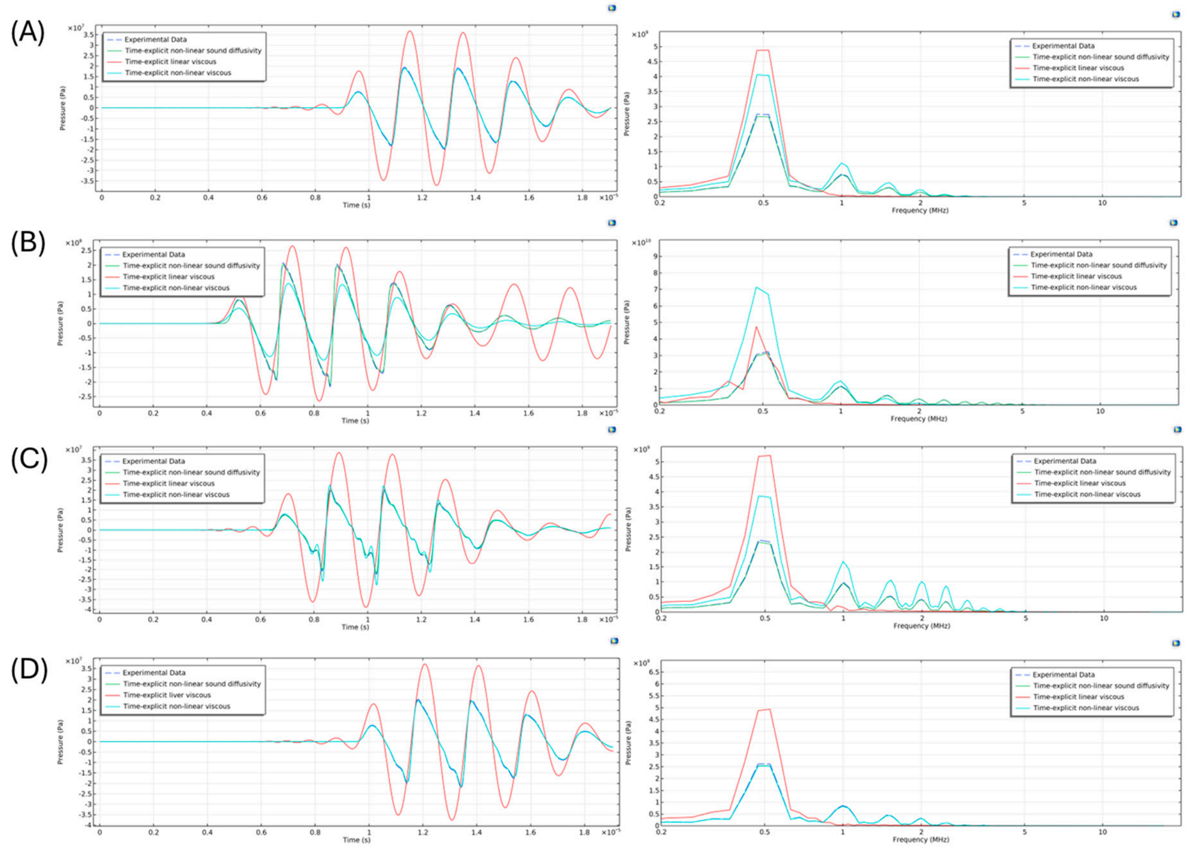

Figure S1*—Comparison of pressure distribution results from numerical model and experimentation using a layer of (a) skin, (b) cortical bone, (c) trabecular bone, and (d) brain muscle.*

The above results are obtained by using a layer of the respective biological tissue layer between the transducer and the receiver in the experimental setup. The measurements were taken using Plane Wave Mitch Transducers. The results from the numerical simulations were obtained using the same parameters and the input sound wave; the comparison was performed by including the results from the work on MATLAB to generate the above figures. These results are explicitly taken from our previous work [59], with the consent of all the authors. Thus, the numerical results show high compliance with the experimental results with respect to the pressure distribution inside the biological tissues. A similar procedure was used to determine the non-linear properties of the tissues through the numerical model used in this work and validated

against experimental results in our previous work [59]. Thus, the numerical model used in this work provides pressure distribution inside biological tissue and respective acoustic properties such as the parameter of non-linearity, speed of sound, coefficient of diffusivity, and coefficient of absorption with significant accuracy. Authors also state that there is a requirement for experimental validation of acoustic wave convergence at different focal positions and the respective acoustic energy due to parametric variations obtained by the 5-layer model human head numerical simulations in this work.

Numerical Simulation Validation through Experimentation

Several time-explicit signals were taken from the simulations used in Chapter 4.2 of the article to analyse the frequency domain, as can be seen in Figure S2.

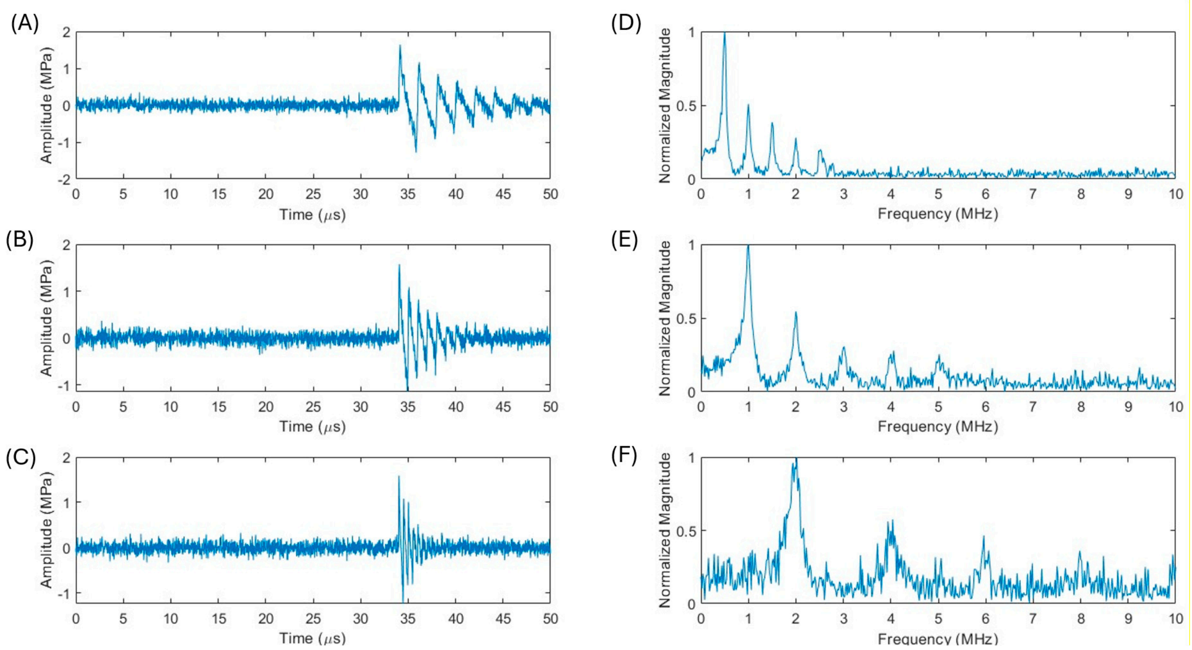

Figure S2—*Signal response captured by the probe in the brain tissue at 45 mm depth in the time-domain at (A) 0.5 MHz, (B) 1 MHz, and (C) 2 MHz and in the frequency domain at (D) 0.5 MHz, (E) 1 MHz, and (F) 2 MHz, where it is possible to observe the higher frequency harmonics typical of biological tissue acoustic wave propagation.*

Figure S2 demonstrates how signal analysis of the signals in the brain revealed higher-order harmonics, demonstrating the simulation is capable of capturing the non-linear behavior of wave propagation, which is a hallmark of acoustic transmission in heterogeneous, viscoelastic media such as brain tissue. Unlike purely frequency-dependent simulations that assume linear superposition and constant attenuation across frequencies, this time-domain approach demonstrates how energy redistributes into harmonic components and gradually diminishes with time. Altogether, this model provides a more realistic depiction of how ultrasound signals evolve in vivo, highlighting the emergence of non-linear effects that cannot be observed when relying solely on frequency-domain attenuation models.
